# Autofluorescence in Plants

**DOI:** 10.3390/molecules25102393

**Published:** 2020-05-21

**Authors:** Lloyd Donaldson

**Affiliations:** Scion, Private Bag 3020, Rotorua 3046, New Zealand; lloyd.donaldson@scionresearch.com; Tel.: +64-7-343-5581

**Keywords:** autofluorescence, chlorophyll, lignin, suberin, phenolic acids, flavonoids, tannins, FLIM, FRET, spectral unmixing

## Abstract

Plants contain abundant autofluorescent molecules that can be used for biochemical, physiological, or imaging studies. The two most studied molecules are chlorophyll (orange/red fluorescence) and lignin (blue/green fluorescence). Chlorophyll fluorescence is used to measure the physiological state of plants using handheld devices that can measure photosynthesis, linear electron flux, and CO_2_ assimilation by directly scanning leaves, or by using reconnaissance imaging from a drone, an aircraft or a satellite. Lignin fluorescence can be used in imaging studies of wood for phenotyping of genetic variants in order to evaluate reaction wood formation, assess chemical modification of wood, and study fundamental cell wall properties using Förster Resonant Energy Transfer (FRET) and other methods. Many other fluorescent molecules have been characterized both within the protoplast and as components of cell walls. Such molecules have fluorescence emissions across the visible spectrum and can potentially be differentiated by spectral imaging or by evaluating their response to change in pH (ferulates) or chemicals such as Naturstoff reagent (flavonoids). Induced autofluorescence using glutaraldehyde fixation has been used to enable imaging of proteins/organelles in the cell protoplast and to allow fluorescence imaging of fungal mycelium.

## 1. Introduction

**Figure 1:**
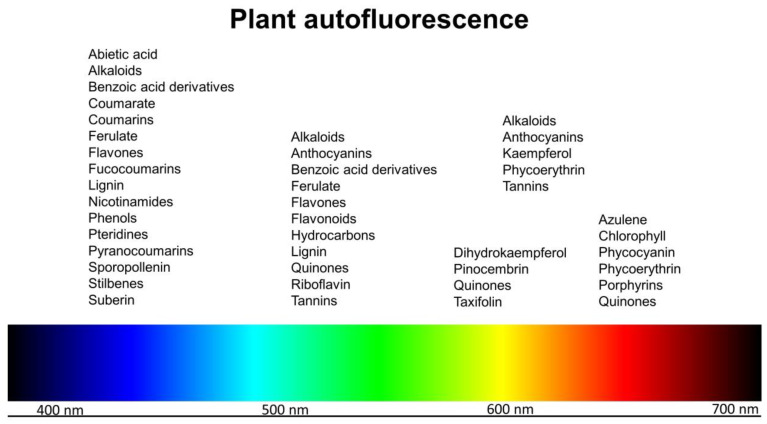
Fluorescence emission of common autofluorescent compounds found in plants [1,2,3,4,5,6,7,8,9,10,11,12,13,14,15].

Autofluorescent molecules are common in plant tissues [1]. This can be viewed from two opposing perspectives. Autofluorescence can be considered problematic [2,3,4]—autofluorescent molecules may interfere with the detection of fluorescent staining, reporter molecules such as GFP (green fluorescent protein), or with immunolocalization techniques. Alternatively, autofluorescence can be used as a label-free method for detecting specific molecules using spectral fluorescence imaging techniques and hence can be considered advantageous for some experimental investigations [1,5,6,7,8,9,10,11,12]. Molecular microscopy using F-techniques (Förster Resonant Energy Transfer (FRET), Fluorescence Lifetime Imaging (FLIM), Fluorescence Recovery after Photobleaching (FRAP), and Fluorescence Correlation Spectroscopy (FCS)) is a growing field of investigation in plant biochemistry, physiology, and pathology and in biomaterial characterization. The two most important autofluorescent molecules found in plants are chlorophyll and lignin but a wide range of other molecules are also autofluorescent with UV or visible excitation including components of both cytoplasm and cell walls (Figure 1) [1].

When investigating autofluorescence in plant tissues, it is important to recognize there may be several types of autofluorescent molecules present at the same location and thus the source of autofluorescence should be interpreted in conjunction with histochemical staining and other forms of analysis [13]. For microscopy applications, autofluorescence should be examined on fresh tissue as some autofluorescent compounds (chlorophyll, flavonoids) can easily become redistributed or be completely removed from tissue when exposed to solvent-based fixatives such as formalin aceto-alcohol (FAA) or mounting media such as glycerol—instead, aqueous buffers can be used as mounting media [14,15].

## 2. Chlorophyll and Other Pigments

**Figure 2:**
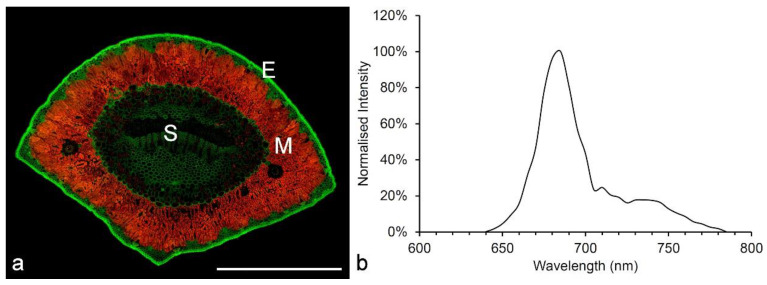
Autofluorescence of chlorophyll in leaves. (**a**) A transverse section (TS) of a pine needle showing autofluorescence of chlorophyll (red) in the mesophyll tissue (M) (excitation at 488 and 561 nm, emission at 500–569, 570–700 nm). The bright green fluorescence in the epidermis (E) originates from flavonoids bound to the cell wall, while the weaker green fluorescence in the stele (S) represents lignin. Scale bar = 500 µm. (**b**) The fluorescence spectrum of pine chlorophyll (excitation at 561 nm, emission at 600–800 nm) [15].

Chlorophyll autofluorescence is used both in lab-scale experiments and in remote sensing applications to evaluate plant health/vigor by directly measuring photosynthetic efficiency and has become a key technique for evaluating the response of plants to changes in the environment [16,17]. Chlorophyll is excited by UV, blue or green light and emits strongly in the red with a distinct bimodal emission with maxima at 685 and 720–730 nm (Figure 2) [16].

**Figure 3:**
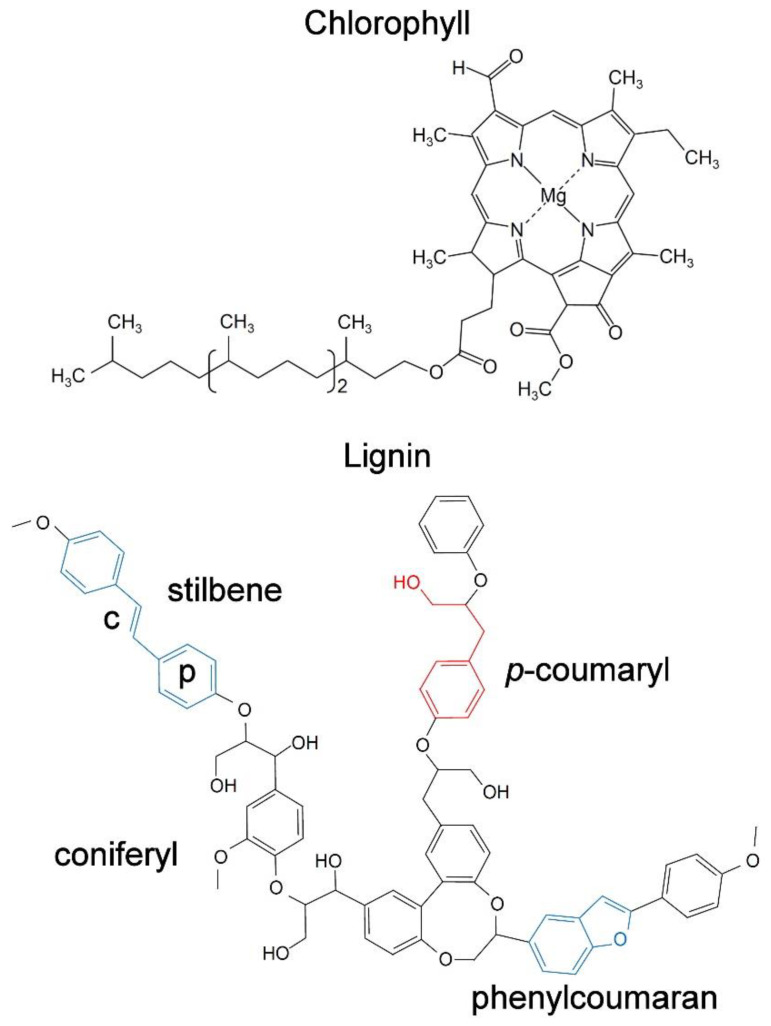
The molecular structure of chlorophyll and lignin to illustrate typical fluorescent structures. Chlorophyll contains a large complex porphyrin ring structure accounting for most of its fluorescence. However, chlorophyll molecules within chloroplasts occur in ordered arrays called photosystems which are also likely to influence the fluorescence. Lignin forms a large complex molecule—only a fraction of which is shown here. Structural components that may contribute to the overall fluorescence of lignin are highlighted including phenolic rings (p) and conjugated bonds (c). These types of structures are characteristic of organic fluorophores.

All chlorophyll molecules possess extensive conjugated bonds in the form of a porphyrin ring that are responsible for the fluorescence (Figure 3) [16]. 

The quantum yield, a measure of the brightness of fluorescence representing the ratio of photons absorbed to emitted, is 0.25 for chlorophyll a in solution and is independent of excitation wavelength or solvent [18]. Because the fluorescence of chlorophyll is a dynamic process in situ, analysis of the spectrum at liquid nitrogen temperatures has been used to obtain more precise spectral information although applications of fluorescence measurement or imaging are usually performed at ambient temperature. Low-temperature spectroscopy has identified a third emission peak at 695 nm [16]. The emission at 685–695 nm is known to be associated with photosystem II, while the broader peak at 720–730 nm is associated with photosystem I [19]. The overall in situ fluorescence of chlorophyll is due to the emission characteristics of both individual chlorophyll molecules and the larger-scale light-harvesting complexes (Figure 3). A characteristic of autofluorescence resulting from multiple emitters within molecules or molecular complexes is the multi-Gaussian model of the emission which can be deconvolved into individual Gaussian (or log-normal) peaks. In the case of chlorophyll, the overall spectrum may contain six Gaussian components with peaks at 680, 685, 695, 700, 720, and 735 nm and these have been associated with various components of photosystem II (680–700 nm) and photosystem I (720–735 nm) [20].

Chlorophyll fluorescence can be used to measure photosynthesis, linear electron flux, and CO_2_ assimilation non-destructively in vivo using commercially available portable sensors [21,22,23,24,25]. This allows for the rapid screening of large numbers of plants to evaluate physiological status in relation to genetic and environmental variables. Although the measurement of fluorescence parameters is simple, several corrections need to be applied related to photochemical and non-photochemical quenching [16]. Chlorophyll content can be assessed at the individual cell level in protoplasts by using autofluorescence and flow cytometry [22].

Anthocyanin pigments extracted from red cabbage leaves show autofluorescence with UV excitation resulting in violet to blue emission. Excitation must be below 460 nm to excite autofluorescence. The extraction of such pigments may result in fractions that have different λ_max_ values that are dependent on excitation wavelength [26]. In *Brassica*, anthocyanins have a λ_max_ in the range of 360–530 nm depending on excitation wavelength (300–410 nm) [26], whereas in *Arabidopsis* the λ_max_ is 680 nm, resulting from multiphoton excitation at 860 nm [27]. Anthocyanins, therefore, have broad and variable autofluorescence emission.

Carotenoids (carotenes and xanthophylls) fluoresce with deep UV excitation, making it difficult to detect them in situ within cellular compartments by microscopy [28]. Carotenoids absorb blue light and transfer this energy to adjacent chlorophyll molecules, thus expanding the range of light energy absorption for photosynthesis [29]. This energy transfer does not involve fluorescence emission although very weak autofluorescence at 560 nm has been reported and attributed to carotenoids in green algae [30] and in the range of 400–500 nm in extracts from Persimmon fruit [28]. 

## 3. Lignin

Lignin is an abundant natural polymer of coniferyl, sinapyl or *p*-coumaryl alcohol that occurs in woody plant tissues, especially in xylem but also in other sclerenchymatous tissues. The interest in lignin autofluorescence is primarily as a means of visualizing lignin microscopically in wood and paper fibers [31]. While there are alternative fluorescent or brightfield stains for lignin, autofluorescence has some advantages from the perspective of increased specificity and label-free imaging as well as in applications for understanding wood nanostructure using techniques such as Förster Resonant Energy Transfer (FRET). Lignin has a characteristically broad emission range due to the presence of multiple fluorophore types within the lignin molecule (Figure 3) and can thus be used with both UV and visible excitation [32,33], providing some flexibility when combined with for example immunohistochemical localization of (non-fluorescent) cell wall carbohydrates such as mannans, xylans and pectins [34]. As with chlorophyll, lignin emission spectra can be deconvolved into multiple log-normal components although these have not been matched to specific fluorescent centers within the complex polymer molecule [35,36]. However, fluorescent model compounds that contain common structures known to occur within lignin polymers have been studied [37,38,39]. Such studies have identified phenylcoumarone and stilbene structures that may represent lignin fluorophores (Figure 3) [37]. Phenylcoumarones as a pure compound in dioxane solution have quantum yields of 0.57–0.61 but the fluorescence intensity of lignin is likely to be much less than this and is strongly dependent on excitation wavelength (37). Lignin fluorescence may also be influenced by the physical structure of the polymer including clustering of carbonyl groups and restrictions on intramolecular rotation [40]. Studies on model compounds have focused on deep UV-excited fluorophores rather than visible wavelength fluorophores in lignin.

Lignin fluorescence is sensitive to the molecular environment and so can be manipulated with pH and by treatment with quenching agents such as nitrophenol-labeled carbohydrates [32,41]. Heat treatment [41,42] and infiltration with modifying chemicals [43] can also influence lignin autofluorescence. Lignin fluorescence can, therefore, be used as a biosensor for various research investigations such as measurement of cell wall porosity or detection of infiltrating chemicals in wood modification studies.

### 3.1. Spectroscopy

**Figure 4:**
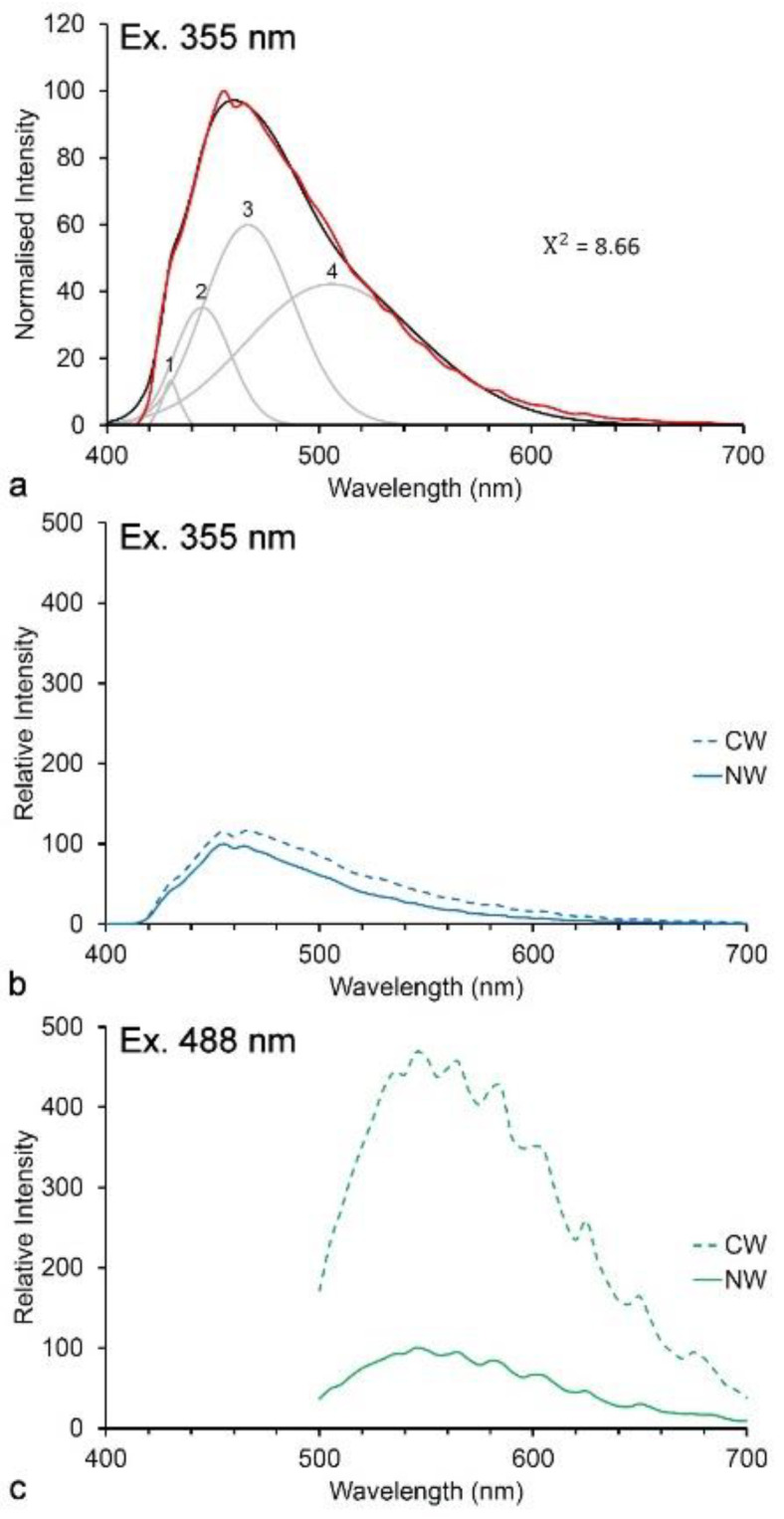
Autofluorescence of lignin in pine wood. (**a**) UV-excited blue fluorescence with λ_max_ at 455–465 nm (excitation at 355 nm, emission at 400–700 nm) showing deconvolution with four log-normal components and a good fit between actual (red) and modeled (black) spectra (Χ^2^ = 8.66, ns). (**b**) Autofluorescence (excitation at 355 nm) of normal (NW) and compression wood (CW) lignin in pine showing a negligible brightness difference. (**c**) Autofluorescence (excitation at 488 nm) of normal and compression wood lignin in pine showing the much brighter green fluorescence reflecting an increased quantum yield in compression wood resulting from blue excitation due to increased *p*-hydroxy lignin units. The blue-excited spectrum shows some evidence of resonance effects (waviness).

Lignin absorbs in the UV range with maximum absorbance at 280 nm [44]. Fluorescence from UV excitation has a peak at 360 nm thought to be due to emission from stilbene and phenylcoumarone structures within the lignin molecule [37]. Fluorescence of lignin in its native state may be quenched due to the presence of covalent linkages to hemicellulose. Treatments that break such bonds including photo-degradation, fungal or bacterial decay, as well as chemical treatments such as removal of carbonyl groups all tend to increase the brightness of lignin fluorescence [33]. However, it is difficult to understand the exact sources of fluorescence in lignin because of the complexity of its structure and its occurrence within a natural composite together with (non-fluorescent) carbohydrates. The extraction of lignin from cell walls inevitably alters its structure and hence its fluorescence. Lignin fluorescence also occurs with visible light excitation suggesting potentially multiple fluorescent structures (Figure 4a) [32,33,35,36]. Both UV and visible emission spectra contain multiple log-normal components resulting from conjugated structures [35,36]. It is quite likely that lignin fluorescence results from resonant energy transfer among different structures [37,40]. When excited by blue laser light, lignin fluorescence shows evidence of weak whispering gallery resonance which may also result from resonant energy transfer processes (Figure 4c) [45,46].

Different lignin types may show small differences in emission spectra (Figure 4b,c). The H-lignin (*p*-hydroxy) found in the middle lamella layer of normal wood and in the secondary wall of compression wood (reaction wood found in leaning stems), contains additional fluorophores, resulting in increased quantum yield at longer wavelengths [35]. While G and S-lignins (guaiacyl and syringyl, respectively) normally do not show any spectral differences, in the special case of S-lignin in poplar which contains *p*-hydroxybenzoate groups, this allows differentiation between G-rich and S-rich cell types [32].

### 3.2. Imaging

**Figure 5:**
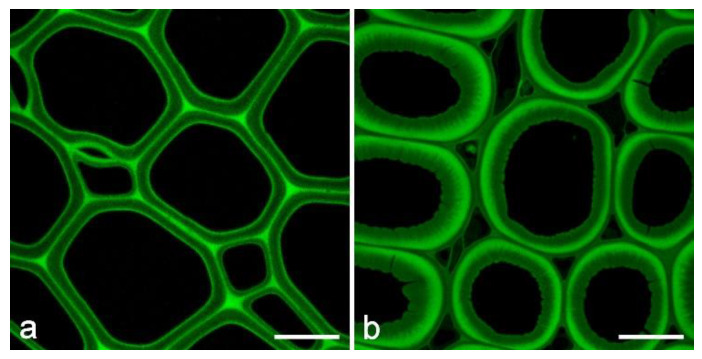
Autofluorescence of lignin in pine wood. (**a**) TS of normal pine wood (excitation at 458 nm, emission at 465–700 nm). Scale bar = 15 µm. (**b**) TS of pine reaction wood showing the altered distribution of lignin (excitation at 458 nm, emission at 465–700 nm). Scale bar = 15 µm.

Lignin fluorescence has been used extensively in imaging studies of wood and paper [31,32]. Using confocal fluorescence microscopy, lignin can be excited with UV (355 nm) or blue (488 nm) light [32]. These two excitations result in slightly different information. The UV-excited emission is similar to that from other phenolic molecules found in plants and has less contrast between the highly lignified middle lamella and less lignified secondary wall of wood fibers. This suggests a stronger influence of evenly distributed chemical groups and less influence of fluorophore abundance and quantum yield. UV-excited lignin fluorescence is, however, more sensitive to the presence of quenching molecules [41]. Blue excitation results in strong contrast between areas of different lignification and is generally much brighter, partly due to the optical properties of the microscope lenses, which makes it more useful for imaging studies (Figure 5) [32]. Lignin autofluorescence is relatively weak compared to detection with lignin stains such as acriflavin but tends to be more specifically related to lignin—acriflavin will stain other cell wall and cytoplasmic (DNA, RNA) components to varying degrees.

Lignin autofluorescence has been used to evaluate wood phenotype in radiata pine genetically modified to downregulate the 4-coumarate CoA ligase (4CL) enzyme in the lignin biosynthetic pathway resulting in reduced and variable lignification of tracheid cell walls [47].

Lignin fluorescence in compression wood has been used as a measure of compression wood severity using both spectral analysis and imaging of lignin distribution [35] and has been used to rapidly screen compression wood distribution within trees [48]. Compression wood has undesirable wood properties compared to normal wood including higher shrinkage and lower axial stiffness.

Lignin autofluorescence has been used for colocalization studies showing the distribution of lignin, relative to hemicelluloses (using immunolocalization) and cellulose (using birefringence) in pine wood [34]. Lignin and mannan were shown to be negatively colocalized in both normal and compression wood whereas lignin and galactan were positively colocalized in compression wood but not in normal wood [34]. Similar studies have shown that, in spruce wood, resin canal parenchyma cells with lignified secondary cell walls do not show a reaction wood response (increased lignin and galactan content) compared to adjacent tracheids [49].

Lignin autofluorescence has been used in several studies to assess cell wall porosity. Lignin fluorescence is quenched in the presence of (non-fluorescent) low molecular weight carbohydrates labeled with a nitrophenol group (e.g., 4-nitrophenyl β-d-glucopyranoside). Thus, similar molecules of different molecular weight can be infiltrated into cell walls and their distribution assessed to determine the relative porosity of different cell wall regions [41]. Similar studies using the FRET interaction between lignin (donor) and rhodamine dye (acceptor) have been used to measure the accessibility of cell wall regions to the dye, demonstrating that highly lignified regions (middle lamella) are less accessible and hence less porous than cellulose-rich regions (secondary cell walls) [50]. These results have been used to optimize methods for the chemical modification of wood cell walls and their infiltration with chemicals used to improve wood properties such as hardness.

Lignocellulosic biomass is a potential source of sustainable biochemicals and liquid biofuels. The presence of lignin inhibits the enzymatic conversion of cellulose by reducing accessibility. There are numerous studies related to the influence of chemical and physical pretreatments designed to mitigate lignin related recalcitrance, many of which use lignin autofluorescence [51,52,53,54,55,56,57,58,59]. In a study comparing the distribution of fluorescently labeled cellulase enzyme to the distribution of both cellulose and lignin in pretreated woody biomass, lignin autofluorescence was used in combination with Congo red staining of cellulose. This study demonstrated a positive spatial correlation between cellulase enzyme and cellulose, and a negative correlation with lignin distribution [56].

## 4. Other Cell Wall-Associated Fluorophores

### 4.1. Ferulate

**Figure 6:**
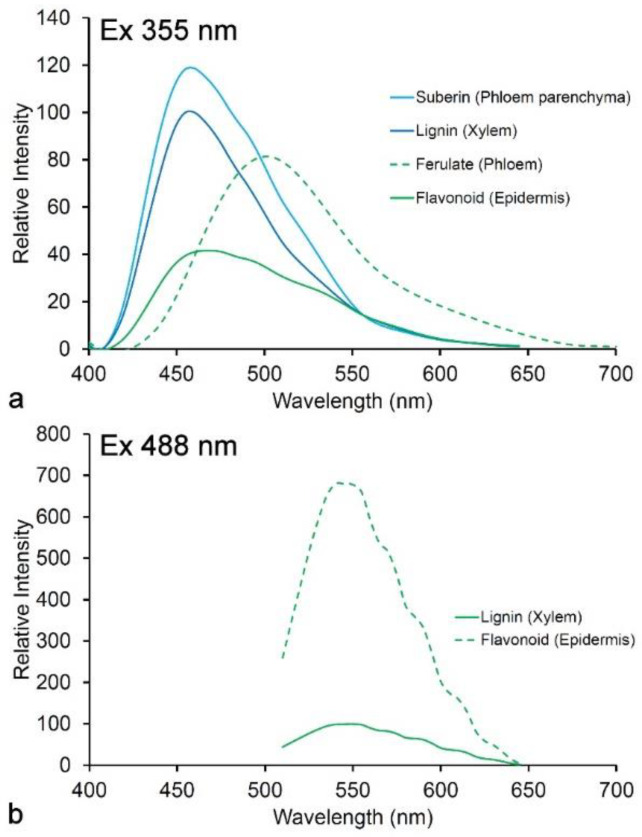
Fluorescence spectra of cell wall autofluorescence in pine needles showing relative brightness. (**a**) Excitation at 350 nm (UV); (**b**) excitation at 488 nm (blue). Flavonoids in the epidermis are much more fluorescent with blue excitation relative to lignin [15].

Ferulic acid may occur in cell walls, where it is usually esterified to polysaccharides, emitting a very weak blue fluorescence with UV excitation at neutral pH that characteristically changes to stronger green emission under conditions of high pH such as in the presence of ammonia (Figure 6) [60]. Ferulic acid is also sensitive to constraint of intramolecular motion, exhibiting an increase in quantum yield (0.0024 to 0.044) when incorporated into rigid biopolymers and hence acting as a biocompatible fluorescent label in biomedical applications [61]. 

In conifers, ferulate can be detected in phloem cell walls, especially in the primary wall/middle lamella, with very weak emission in the secondary wall [15,60,62]. There are no histochemical tests for ferulate, so its identification in tissue is based solely on demonstrating a pH-dependent change from blue to green fluorescence. Other phenolic acids may also produce weak UV-excited blue autofluorescence in cell walls.

### 4.2. Cutin

The cuticle of plants shows autofluorescence attributed to cutin. The cuticle located on the outer surface of the epidermal cell wall may be of variable thickness among species, only 20–50 nm in *Arabidopsis*, and hence may be difficult to distinguish from the often highly fluorescent epidermal cell wall which is usually very thick (several micrometers). Cutin has a similar fluorescence spectrum to suberin and lignin [63,64,65]. Studies of cutin-deficient mutants in tomato have been carried out but, unfortunately, autofluorescence was not examined [64]. The exact nature of cutin fluorescence is poorly understood. Cutin is a polyester-based material [66], and fluorescence may originate from phenolic acids or flavonoids bound to the cutin backbone similar to the fluorophores present in the adjacent epidermal cell wall (Figure 6) [15,65].

### 4.3. Suberin

Suberin, like cutin, is a hydrophobic polyester consisting of long-chain fatty acids and glycerol [67]. It exhibits UV-excited autofluorescence comparable to lignin, from which it can be distinguished by its weak blue-excited autofluorescence relative to lignin (Figure 6) [15,49]. In sequentially UV/blue-excited images, suberin will show violet/blue emission, while lignin will show blue/green emission, although both lignin and suberin may be colocalized and hence indistinguishable in some tissues (endodermis, epidermis, Casparian strips).

### 4.4. Sporopollenin

**Figure 7:**
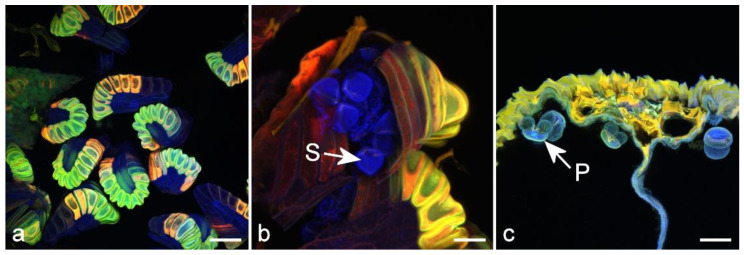
Autofluorescence in reproductive structures. (**a**) Immature fern sporangia. Scale bar = 100 µm. (**b**) Mature sporangium with spores (S). Scale bar = 33 µm. (**c**) TS of a male cone in pine with mature pollen grains (P). Scale bar = 50 µm.

Sporopollenin is a chemically inert biopolymer found in the outer layer of spores and pollen. It is composed of aliphatic-polyketide-derived polyvinyl alcohol units and 7-*O*-*p*-coumaroylated C16 aliphatic units [68]. The outer wall of pollen grains and fern spores is autofluorescent, with emission at both blue and yellow wavelengths assumed to be due in part to sporopollenin [69], although other potential fluorophores including pigments such as carotene, flavonoids such as naringenin and terpenes such as azulene may also be present [70]. The autofluorescence is known to be variable both during development and between different species [71,72]. Fluorescence spectra of pollen and spores show indications of three fluorescent peaks, at 475, 565, and 675 nm [72]. The consensus seems to be that the UV-excited blue fluorophore in fern spores and pollen grains is sporopollenin while green/yellow fluorescence may be flavonoids or pigments (carotenoids, lipofuscin), but this needs further study, reflecting the complex mixture of potential fluorophores that may be present (Figure 7).

### 4.5. Flavonoids

Plants contain a wide range of different flavonoids that may be associated with cell walls or may occur as secretions or extractives. A fluorescent flavonoid known as *Lignum nephriticum* (matlaline), the oxidation product of flavonoid extractives from *Eysenhardtia polystachya*, a tropical tree, was first described in the sixteenth century [73]. Flavonoids are of interest as potential therapeutic agents and are autofluorescent with emission at green, yellow and orange wavelengths. This fluorescence can be specifically enhanced by using a reagent containing 2-aminoethyl-diphenylborinate (Naturstoff reagent—DPBA) due to complex formation between DPBA and the flavonoid molecule [74].

Flavonoids are biologically active molecules that occur in plants in more than 10,000 structural variants. Flavonoids have important roles in auxin transport, development of roots and shoots, control of reactive oxygen species, pollination, symbiotic nodule formation, and as defense compounds [75]. 

Flavonoids (as flavonol glucosides), together with hydroxycinnamic acids and other weakly autofluorescent phenolic materials, are responsible for the fluorescence of the leaf epidermis (Figure 2 and Figure 7) [15,63,76,77].

The autofluorescence properties of flavonoids from paprika have been studied by spectrofluorimetry [78]. Fluorescence intensity was found to increase in high pH medium and multivariate analysis was used to determine optimum excitation and emission characteristics of myricetin, quercetin, and kaempferol. Optimal excitations were in the range of 460–480 nm, with blue and green emission.

The role of flavonoids in auxin transport, gravitropism, and phototropism in mutants of *Arabidopsis* has been investigated using autofluorescence to localize kaempferol, quercetin, and naringenin in root tissues [79,80]. Both kaempferol and quercetin were localized to the nucleus and endomembrane systems of hypocotyl cells.

**Figure 8:**
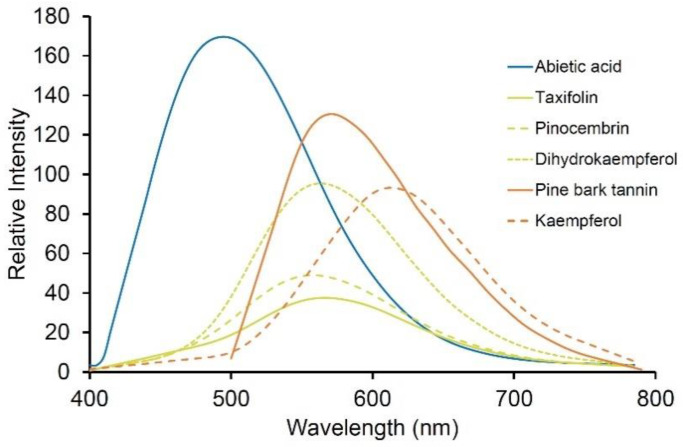
Fluorescence spectra of some common purified wood extractives (from Sigma except for pine bark tannin manufactured by Scion) showing relative brightness [14]. Excitation was at 355 nm except for pine bark tannin which was excited at 488 nm.

Flavonoids including dihydrokaempferol, pinocembrin, and taxifolin are major components of extractives in the wood of Douglas-fir occurring within heartwood tracheid cell walls and in resin canals [14]. The fluorescence of extractive deposits has been compared with purified standards allowing identification of different autofluorescent extractive deposits in resin canals and rays (Figure 8).

## 5. Other Fluorophores

### 5.1. Stilbenes

Purified, piceid, pterostilbene and resveratrol show violet emission, with a λ_max_ of 400 nm in water at neutral pH and blue emission at 450–470 nm at high pH. Stilbenes in grape berries were imaged with UV excitation and blue emission and their presence in epidermal vacuoles was associated with reduced susceptibility to downy mildew [81].

### 5.2. Tannins

Condensed tannins are polymers of catechins generally known as proanthocyanadins. They occur as abundant orange fluorophores in bark and wood (λ_max_ = 565 nm) [82,83] and may also occur in other plant tissues such as leaves and fruit, with fluorescence emissions over the range of 500–650 nm (Figure 8) [84,85]. 

### 5.3. Terpenes

The fluorescence of terpenes is variable, with some common terpene extracts such as pinene and limonene being non-fluorescent. Likewise, the triterpene waxes on the surface of fern fronds are non-fluorescent. The exact chemical nature of fluorescence in plant secretory compounds requires more attention as these are more than likely mixtures when they occur in situ within the plant. Azulene is a fluorescent terpenoid that occurs in the secretory cells of plants with emission at red wavelengths [1,10]. This compound is notable as the only known example where fluorescence arises from an upper excited state (S2 → S0) [86]. Resin acids such as abietic acid, a diterpene, are known to be autofluorescent with UV excitation and blue emission (Figure 8). Oleoresins are also weakly autofluorescent with blue emission [14,15]. 

## 6. Induced Autofluorescence Using Glutaraldehyde

**Figure 9:**
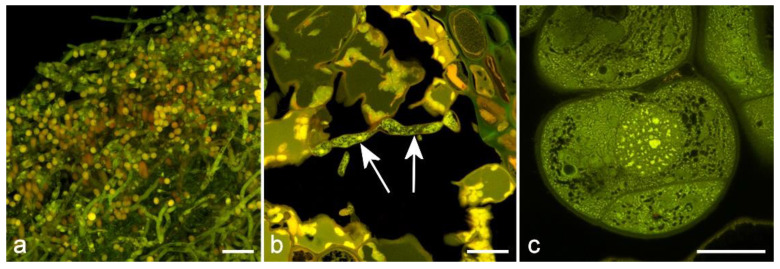
Glutaraldehyde-induced autofluorescence of plant tissues. (**a**) TS of *Penicillium* in culture showing hyphae and spores. Scale bar = 10 µm; (**b**) Pine needle infected with *Phytopthora pluvialis* (arrows). Scale bar = 20 µm. (**c**) Pine somatic embryo using Super-Resolution Radial Fluctuation (SRRF) microscopy [90], showing dense cytoplasm with mitochondria, endoplasmic reticulum, nucleoli, and Golgi vesicles. Scale bar = 10 µm.

Treatment of plant tissue with glutaraldehyde will induce or enhance autofluorescence of proteins, so it is a useful method for imaging of cytoplasm in fixed tissue (Figure 9) [63,87,88]. While for some applications this might be problematic, glutaraldehyde fixation can produce excellent images of cytoplasm and can be used for high-resolution confocal fluorescence imaging as opposed to techniques like DIC or phase contrast which are limited to widefield microscopy. Typically, this approach produces a bland yellow autofluorescence (λ_max_ 560 nm) with little or no spectral differentiation but can potentially be combined with fluorescence from other natural fluorophores present within the tissue, or with blue or red-fluorescent dyes for multiple labeling experiments. This method can be particularly useful for imaging fungal hyphae which are difficult to label with fluorescent stains (Figure 9). Fungal hyphae and spores may also show some natural autofluorescence [89].

## 7. Deep UV Excitation

Some autofluorescent compounds absorb and emit at shorter wavelengths below the visible part of the spectrum—amino acids such as tryptophan, for example. While measuring fluorescence spectra of materials extracted from plants is straightforward at these excitation wavelengths, in situ spectroscopy and imaging are difficult at deep UV excitation wavelengths due to the lack of suitable lasers (for confocal fluorescence imaging) as well as limitations in the characteristics of lenses. Deep UV fluorescence images will be heavily shaded—that is, the emission may be uneven due to inhomogeneity of illumination at short wavelengths. Nevertheless, some imaging studies have been performed on plant tissues in situ using synchrotron radiation for excitation to localize protein (cellulase enzyme) and phenolic residues [91,92].

## 8. Spectral Imaging and Unmixing

### 8.1. Sequential Excitation/Emission Imaging

**Figure 10:**
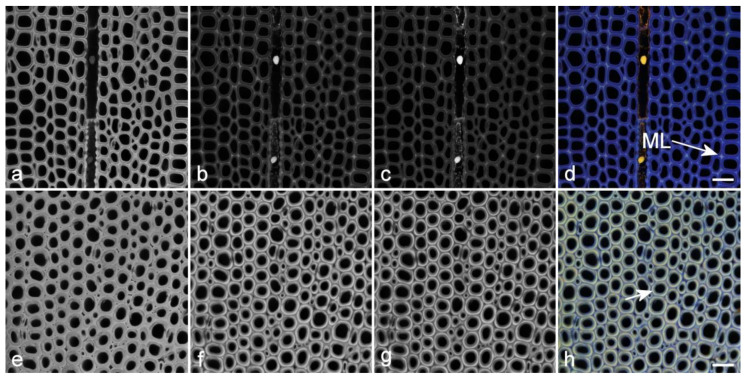
Spectral imaging of lignin autofluorescence using sequential excitation with emission ranges centered around the λ_max_ for each excitation wavelength at a constant gain. Excitation at 355 nm (UV) (**a**,**e**), 488 nm (blue) (**b**,**f**) and 561 nm (green) (**c**,**g**). Color overlays are shown in (**d**,**h**). (**a**–**d**) Normal pine wood showing monochromatic lignin fluorescence at UV, blue and green excitations. Scale bar = 30 µm. (**e**,**h**) Pine compression wood showing the same range of excitation and emission highlighting the change in lignin distribution in this wood type compared to normal wood. UV excitation shows the same intensity, whereas blue and green excitations are significantly brighter compared to normal wood (images of normal and compression wood were acquired at the same gain). Blue and green excitation clearly show the more lignified middle lamella (ML) and outer secondary cell wall (short arrow) in the two specimens respectively. Scale bar = 30 µm.

**Figure 11:**
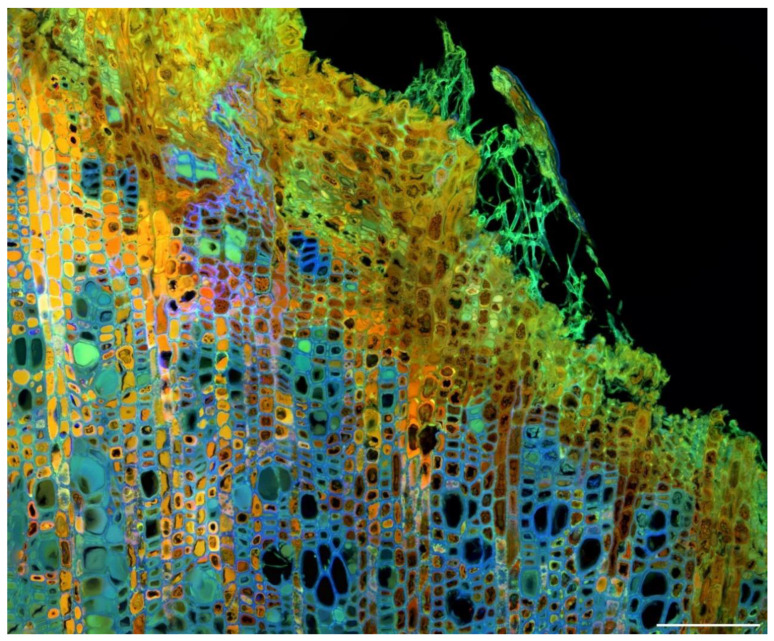
Spectral imaging of autofluorescence in a diseased apple stem (*Nectria* canker) using sequential excitation with emission ranges centered around the λ_max_ for each excitation wavelength. Excitation at 355 nm (UV), 488 nm (blue), and 561 nm (green). Many different unidentified fluorophores are present. However, blue cell walls represent lignin. Scale bar = 100 µm.

Since plant tissues may contain multiple autofluorescent compounds, it is often of interest to produce images that show their relative locations [14,15]. Imaging with a single excitation and a single emission band only detects total fluorescence as well as spatial variation in intensity (Figure 5). This provides limited information on the spatial distribution of multiple fluorophores. Instead, using one or more excitation wavelengths and multiple emission bands will produce a color image by assigning blue, green, and red to the emission bands, providing some spectral separation of potentially many different fluorophores (Figure 10 and Figure 11) [63]. The most effective way of doing this is to sequentially acquire each emission band using only a single excitation wavelength at a time. On filter-based fluorescence microscopes, this is very slow because of the need to physically move filter wheels and dichroic mirrors during the acquisition. However, AOTF/AOBS-based systems (acousto-optical tunable filter and acousto-optical beam splitter) electronically change the excitation/emission signals, allowing sequential acquisition line by line in real-time rather than the tedious frame by frame serial acquisition required for filter-based instruments. Sequential imaging allows potential overlap of excitation and emission bands and eliminates bleed-through between emission bands. Bleed-through occurs when emission from a shorter wavelength excitation is detected in the emission channel of a second longer wavelength excitation when the two excitations are applied simultaneously. This can easily be demonstrated by removing the shorter wavelength excitation and observing a reduction in brightness of the longer wavelength emission channel.

### 8.2. Unmixing

**Figure 12:**
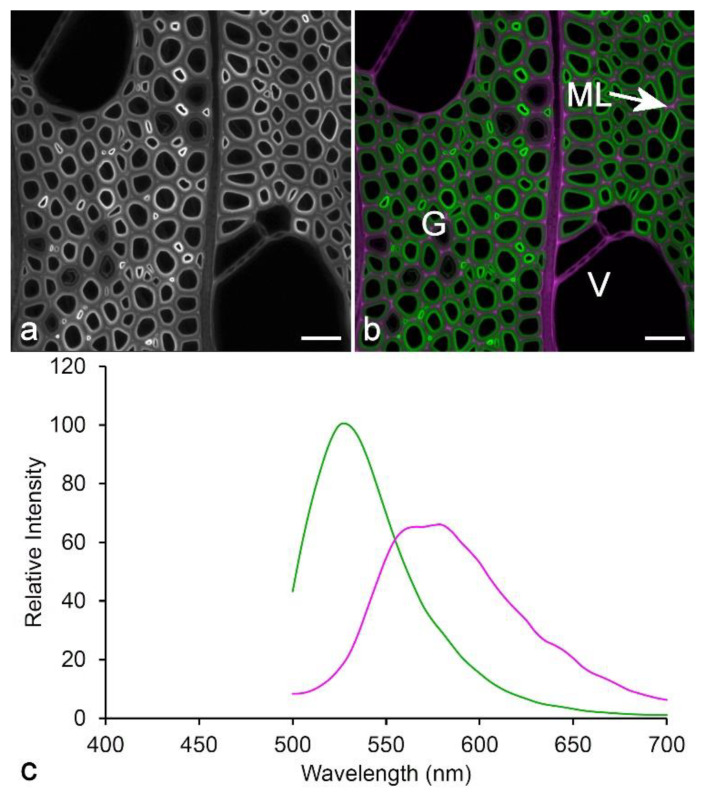
Spectral unmixing using Poisson NMF in ImageJ software [93]. (**a**) Poplar wood showing monochromatic lignin fluorescence (excitation at 488 nm, emission at 500–700 nm). Scale bar = 30 µm. (**b**) Poplar wood showing two-channel spectral unmixing calculated using Poisson NMF from a spectral image sequence (excitation at 488 nm, emission at 500–700 nm in 5 nm steps). The crimson regions correspond to areas rich in guaiacyl lignin (vessel cell walls [V] and middle lamella [ML]), while the green regions are rich in syringyl lignin (fiber secondary cell walls). The darker fibers (G) are gelatinous fibers (reaction wood) with weakly lignified cell walls. Scale bar = 30 µm. (**c**) Spectra calculated by blind unmixing using Poisson NMF [32,93]. The use of green and crimson for two-channel overlays is an alternative to green and red to accommodate color-blind readers.

An alternative method to spatially localize multiple fluorophores in plant tissue is to perform spectral unmixing on a spectral sequence (a series of images acquired at small wavelength intervals using a single excitation wavelength) (Figure 12). This can be done either by using standard reference spectra or by using blind deconvolution algorithms [93], and in practice, these give essentially identical results. This usually works best with only a single excitation wavelength although sequential lambda sequences can be combined. This can potentially allow identification and localization of multiple fluorophores of the same color—two green fluorophores, for example, provided they have slightly different spectral characteristics. This technique has been used to localize different lignin types in poplar wood, where the fluorophores both have green emission with small differences in λ_max_ and relative intensity [32]. 

Because chlorophyll fluorescence can interfere with imaging of expression tags such as fluorescent proteins, or other stains including immunolabeling, spectral unmixing can be used to isolate the chlorophyll fluorescence, allowing more precise localization of the tag or stain [94].

## 9. F-Techniques Using Autofluorescence

### 9.1. Fluorescence Lifetime Spectroscopy (FLIM)

FLIM offers an alternative to fluorescence intensity as a way of imaging the autofluorescence of plant tissues. Fluorescence lifetime (τ_m_) is the time in picoseconds or nanoseconds between excitation of the fluorophore and its subsequent emission and is usually measured using time-correlated single-photon counting (TCSPC) [95].

Multiphoton FLIM has been used to study secondary metabolites in *Eucalyptus* secretory cavities [96]. Because sectioning results in damage to cells and subsequent loss of vacuolar contents, the use of multiphoton microscopy with its greater depth penetration allowed imaging of cellular contents up to 80 µm into the secretory structures. Secretory cavities contained autofluorescent oleuropeic acid glucose esters (τ_m_ = 1.4–1.5 ns) as well as non-fluorescent essential oils. Cell walls contained autofluorescent components with much shorter lifetimes—possibly ferulate or flavins.

Fluorescence lifetime imaging has also been used to detect anthocyanins in different cellular compartments using an anthocyanin-free mutant as a negative control [27]. Anthocyanins located in vacuoles had shorter lifetimes compared to those in the cytoplasm and this was associated with a lower pH in the vacuole [27]. 

Fluorescence lifetime has been used in many studies on chlorophyll fluorescence and photosynthesis in algae and in higher plants [97,98]. In *Chlamydomonas*, changes in chlorophyll fluorescence lifetime were observed in zeaxanthin-accumulating mutants associated with the photoprotection function of these pigments. Zeaxanthin pigments quench chlorophyll fluorescence in a process that probably involves FRET [99,100]. Measurements of lifetime have been used to understand the various components of chlorophyll fluorescence in relation to photosystems I and II and to assign such components to fluorophore structures within the chlorophyll molecule [101]. Other applications include assessment of disease states [102], desiccation [103], and heavy metal toxicity [104].

**Figure 13:**
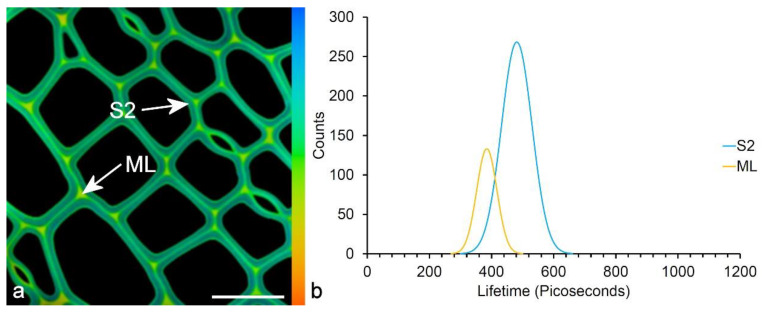
Fluorescence lifetime of pine lignin. (**a**) TS of pine wood showing a difference in fluorescence lifetime between the middle lamella (ML) and the secondary wall (S2). (Excitation at 405 nm.) Scale bar = 30 µm. Lifetime color scale from 0 to 1000 ps. (**b**) Fluorescence lifetime spectrum of the middle lamella (ML) compared to the secondary cell wall (S2) showing a shorter lifetime [105].

Imaging and spectroscopy have been used to determine the fluorescence lifetime of wood lignin and to study its variation between cell wall layers and among wood types [105]. Lignin has a relatively short lifetime in the range of a few hundred picoseconds (Figure 13). There is a unimodal spectrum in normal wood and a bimodal spectrum in compression wood, which reflects the distribution of H-lignin in the middle lamella of normal wood and in the secondary wall of compression wood. The differentiation of G and H-lignins is somewhat greater by lifetime compared to intensity. Changes in lignin composition based on CAD mutants in *Arabidopsis*, where alcohol monomers are replaced with aldehydes have been shown to result in a change in fluorescence lifetime correlated to changes in composition determined by Raman spectroscopy [58]. Fluorescence lifetime has also been used in studies of pretreated woody biomass where changes in lifetime can be related to chemical pretreatments that influence amenability to enzymatic digestion for biofuel production [54,57]. FLIM has a lot of potential for plant autofluorescence applications, as it can potentially reveal differences that are much less obvious or not apparent in fluorescence intensity images. Lifetime is independent of intensity.

### 9.2. Förster Resonance Energy Transfer (FRET)

FRET is a method for measuring molecular interaction between two fluorophores with overlapping excitation and emission spectra. FRET occurs when a donor fluorophore and an acceptor fluorophore are in close proximity (<10 nm), making possible a non-radiative transfer of excitation energy from the donor to the acceptor. Typically, this technique is used to measure interaction between two labeled proteins where the labels form a FRET pair [106]. FRET is measured by determining the change in fluorescence intensity of the donor and/or acceptor, or the change in fluorescence lifetime of the donor in the presence or absence of the acceptor. Autofluorescence may be a hindrance to studies involving FRET between dyes or endogenous expression tags such as GFP in plant cells [94,107]. Chlorophyll is especially problematic due to its bright fluorescence and wide range of excitation [94].

FRET between an autofluorescent donor and a dye-based acceptor has been described for wood and wood-based biomaterials [14,50,52,55]. Lignin and rhodamine form a FRET pair, with lignin as donor and rhodamine as acceptor, as determined by acceptor photobleaching [55] and FLIM [57]. Lignin autofluorescence with 458 nm excitation does not contribute any bleed through to emission from rhodamine excited at 561 nm and hence the two fluorophores can easily be distinguished, rhodamine fluorescence being very much brighter than lignin autofluorescence in the same emission range. FRET has been used to measure the accessibility of cell walls to rhodamine dye either when the cell wall is infiltrated with extractives as in heartwood [14], or when cell wall-based biomass has been pretreated to increase its porosity for enzymatic conversion of cellulose [52,55]. These studies have shown that lignin is less accessible to rhodamine in cell walls infiltrated with heartwood extractives [14]. More lignified regions of the cell wall (middle lamella) are less porous than those that are less lignified (secondary wall) [50,55]. There are some indications that lignin autofluorescence and extractive autofluorescence can also interact by FRET [14].

### 9.3. Colocalization

Colocalization can involve the use of various statistical measures to quantitatively assess the tendency for fluorophores to be at the same locations in tissues [108]. Lignin autofluorescence has been used to colocalize lignin with other cell wall components such as hemicelluloses detected by immunolocalization [34] or with fluorescently labeled enzymes for biotechnology applications [56].

## 10. Conclusions

Plants contain a wide array of autofluorescent molecules such as pigments, secretory compounds, or structural components of cell walls. Autofluorescence has applications in measuring the physiological status of crops or experimental plots based on measurements of chlorophyll fluorescence, and in imaging studies with applications in plant biochemistry, physiology, pathology, wood science, and plant biotechnology. Combining autofluorescence, including both intensity and lifetime imaging and spectroscopy, with other spectroscopic techniques such as Raman microspectroscopy and Fourier transform infra-red microspectroscopy (FTIR microscopy) may allow more accurate characterization of tissue fluorescence in plants at the cellular level in future studies [13,58]. Applications for using autofluorescence in techniques such as FRAP and FCS, which are used to track the diffusion of molecules in cells, may be forthcoming in the near future.
